# Supplementary figures and images for: Short Peptides of Innate Immunity Protein Tag7 (PGLYRP1) Selectively Induce Inhibition or Activation of Tumor Cell Death via TNF Receptor
Source: Int J Mol Sci. 2023 Jul 12;24(14):11363. doi: 10.3390/ijms241411363 (PMC10379010; doi:10.3390/ijms241411363)

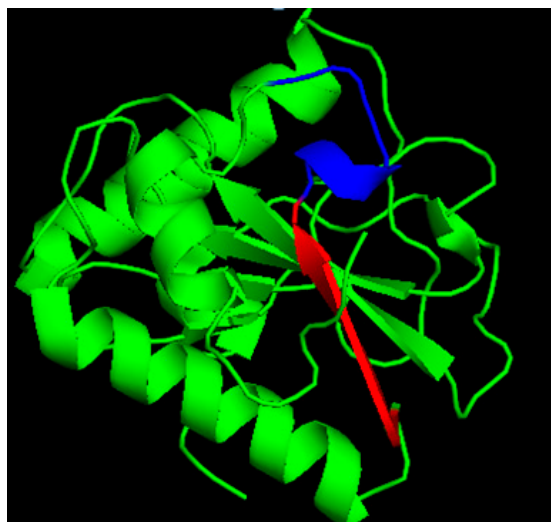

Figure S1. 3D-structure of Tag7 (PGLYRP1, PDB-1YCK) protein. 17.1A – red, 17.1B – blue

Supplement: Supplementary file 1 [file ijms-24-11363-s001.zip › ijms-2491003-supplementary.pdf]
